# Radiation recall dermatitis with soft tissue necrosis following pemetrexed therapy: a case report

**DOI:** 10.1186/1752-1947-3-93

**Published:** 2009-11-02

**Authors:** Christian Spirig, Aurelius Omlin, Giannicola D'Addario, Klaus-Dieter Loske, Philipp Esenwein, Jan Henning Geismar, Thomas Ruhstaller

**Affiliations:** 1 Department of Oncology-Hematology, Cantonal Hospital, St.Gallen, Switzerland; 2 Oncological Palliative Medicine, Cantonal Hospital, St.Gallen, Switzerland; 3 Department of Dermatology, Cantonal Hospital, St.Gallen, Switzerland; 4 Department of Plastic-Surgery, Cantonal Hospital, St.Gallen, Switzerland; 5 Department of Radiation Therapy, Cantonal Hospital, St.Gallen, Switzerland

## Abstract

**Introduction:**

Radiation recall dermatitis is a well known but still poorly understood inflammatory reaction. It can develop in previously irradiated areas and has been shown to be triggered by a variety of different drugs, including cytostatic agents. Pemetrexed may cause radiation recall dermatitis in pre-irradiated patients.

**Case presentation:**

We present the case of a 49-year-old Caucasian woman with non-small cell lung cancer who was initially treated with carboplatin and paclitaxel concomitant with radiotherapy after suffering a painful plexus brachialis infiltration. Due to disease progression, a second-line treatment with pemetrexed was started. A severe soft tissue necrosis developed despite steroid treatment and plastic surgery.

**Conclusion:**

To the best of our knowledge, we present the first case of a patient with severe soft tissue necrosis in a pre-irradiated area after pemetrexed therapy. We believe that physicians treating patients with pemetrexed should be aware of the severe, possibly life-threatening effects that may be induced by pemetrexed after previous radiation therapy.

## Introduction

Radiation recall dermatitis is a well known but still poorly understood inflammatory reaction that can develop in previously irradiated sites, and can be triggered by a variety of different drugs, including cytostatic agents such as gemcitabine, taxanes or anthracyclines [1-3]. Two case reports of radiation recall dermatitis after administration of pemetrexed have been published, but neither caused extensive soft tissue necrosis [4,5]. The most frequent site of radiation recall is the skin, but radiation recall reactions have also been reported to affect the brain, the lungs and the gastrointestinal-system [6-8]. The interval between radiation therapy and onset of radiation recall dermatitis is variable, however, more severe reactions have been described towards the completion of radiation therapy [9]. Radiation recall dermatitis may also develop without clinically apparent prior radiation toxicity. The aetiology of radiation recall dermatitis is still unknown. It is normally a self-limiting reaction without long-term sequelae.

## Case presentation

A 49-year-old Caucasian woman presented with stage IV squamous cell lung cancer (cT4 N3 M1, multiple pulmonary lesions). She had a smoking history of 20 pack years. Palliative chemotherapy was initiated with carboplatin AUC 3 and paclitaxel 75 mg/m^2^, on days 1, 8 and 15, and repeated every 28 days. Radiation therapy for painful left-sided plexus brachialis infiltration with partial arm paresis was performed concurrently during cycle two and three. A total dose of 39 Gy was administered in 13 fractions with 6× photons over a total treatment time of 2.5 weeks. A conformal plan with equally weighted opposing fields from antero-posterior and postero-anterior was used. During radiotherapy, the patient developed typical symptoms of radiation dermatitis. The initially dry maculopapular rash and pruritus worsened one week after completion of radiotherapy to National Cancer Institute Common Toxicity Criteria (NCI-CTC) grade 3 with erythema and small ulcerations in the medio-scapular and cervical left radiation fields. The acute dermatitis resolved within three weeks after application of local and systemic steroids. The subsequent chemotherapy cycles did not cause any further dermal problems and resulted in a partial response after a total of six cycles.

Three months later, the pulmonary metastases progressed and a new adrenal mass was detected on her right side. Second-line chemotherapy with pemetrexed (Alimta, multitargeted antifolate, Eli Lilly) was proposed. The first pemetrexed dose of 500 mg/m^2 ^was given eight months after completion of radiation therapy with standard pre-medication of folic acid, vitamin B12 and dexamethasone. After the first dose, the patient reported increasing dysaesthesia in the former radiation area, however, no skin reaction was detected on clinical examination. The second dose of pemetrexed, given three weeks later, led to massive dermatitis one week after the second infusion in the area of the former radiation field, strictly restricted to the back and sparing the front side of the radiation field. Direct tumour infiltration of the skin was excluded by skin biopsy. The diagnosis of radiation recall dermatitis was suggested and pemetrexed was stopped.

**Figure 1:**
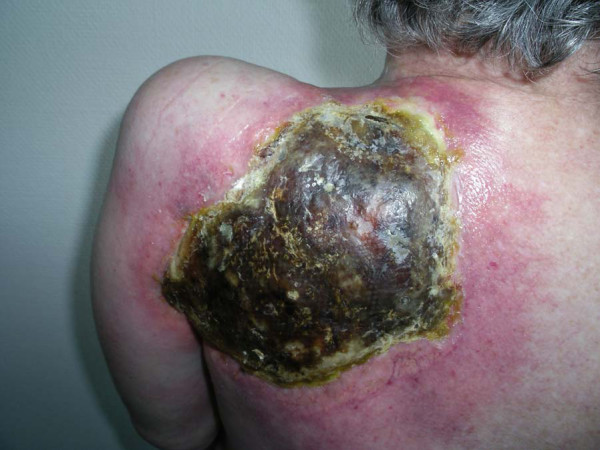
**Extensive dermal necrosis in the pre-irradiated scapular region about three weeks after the second dose of pemetrexed**.

**Figure 2:**
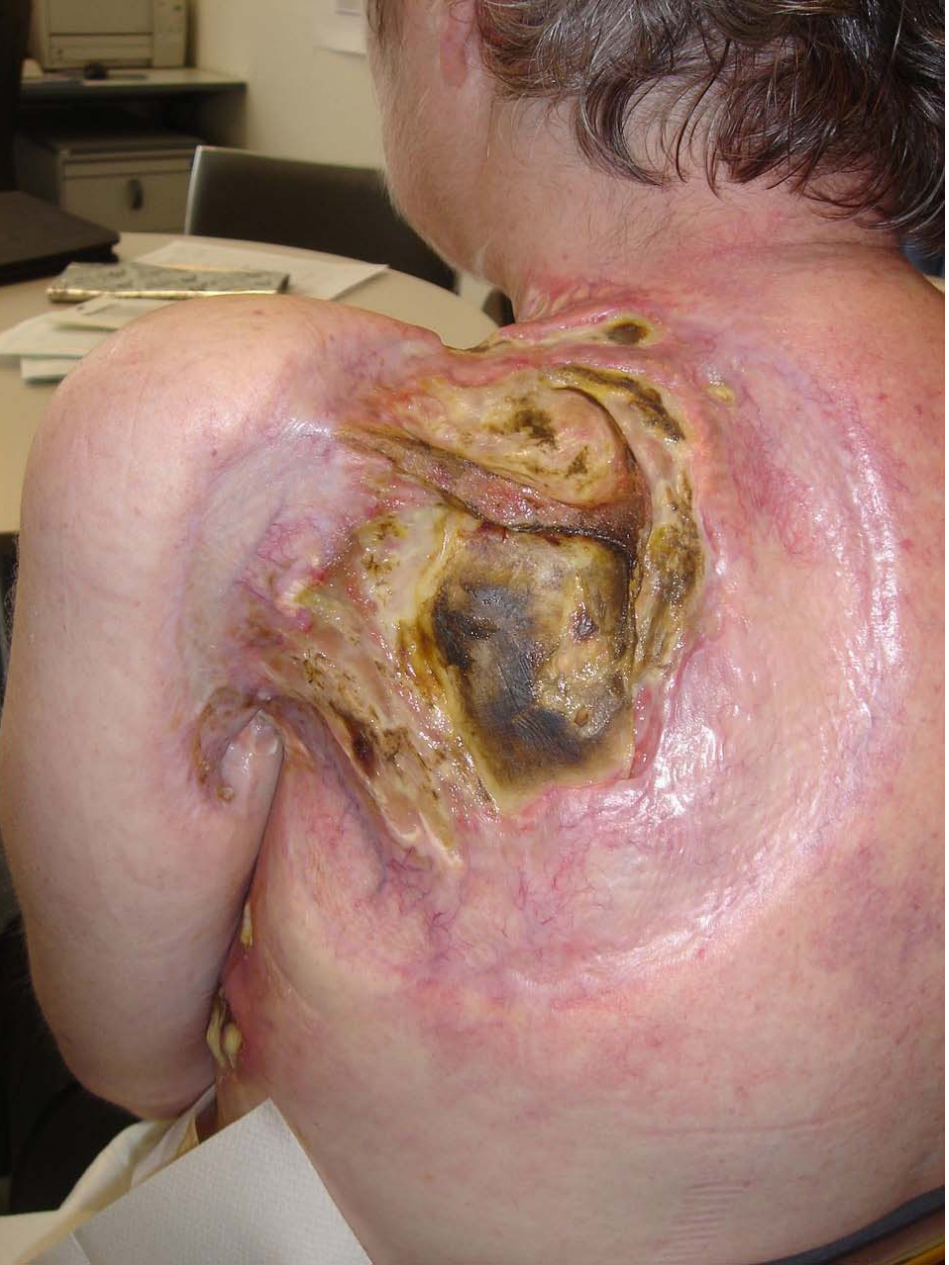
**Ongoing destruction in the pre-irradiated area with infiltration of the scapula about nine months after pemetrexed**.

Despite immediate local and systemic treatment with steroids (60 mg of prednisone) and antibiotics (amoxicillin), a progressive necrosis of the skin and underlying soft tissue developed over the following two weeks (Figure 1) causing massive pain and immobilisation of the left shoulder and arm. A surgical debridement of the necrosis and a musculus latissimus dorsi flap from the contralateral side as well as additional primary coverage with a cutaneous mesh craft were performed. The muscle flap became necrotic and in a second operation, the flap had to be removed. The large tissue defect showed no signs of improvement despite intensive local wound care. On the contrary, continuous destruction occurred with involvement of the scapula (Figure 2). Due to the ongoing necrosis with deteriorating condition of the patient, we abstained from further chemotherapy. Sixteen months after the chemotherapy had been stopped, the patient died of uncontrolled local infection and concurrent pneumonia.

## Discussion

This case demonstrates unexpected and extremely severe radiation recall dermatitis with soft tissue necrosis. Pemetrexed was the probable cause as this was the only new drug used at the time of the developing radiation recall dermatitis. There are two known previous cases with much less severe radiation recall dermatitis in patients treated with pemetrexed [4,5]. In the first case, the patient had been treated 25 years before with adjuvant radiotherapy for breast cancer before receiving second-line treatment with pemetrexed for a non-small cell lung cancer (NSCLC). In the other case, the patient had received cisplatin and pemetrexed after having received radiotherapy with 21 Gy for a mesothelioma. Indeed our patient had received a higher dose of radiation therapy (39 Gy versus 21 Gy) and the interval between radiation therapy and exposure to pemetrexed was shorter (8 months versus 25 years) than in the abovementioned cases. This may serve as a possible explanation for the massive reaction we observed.

## Conclusion

Pemetrexed is becoming increasingly used in second-line treatment for NSCLC [5], often in patients with prior radiation therapy. We therefore believe that physicians treating patients with pemetrexed need to be aware of this severe treatment-caused complication. After the first dose of pemetrexed, the patient described slight discomfort with dysaesthesia, and after the second dose, the full-blown clinical symptoms developed. Not only careful examination and assessment by experienced clinicians but also the awareness of recall dermatitis due to pemetrexed may prevent new cases occurring.

## Consent

Written informed consent was obtained from the patient's family for publication of this case report and any accompanying images. A copy of the written consent is available for review by the Editor-in-Chief of this journal.

## Competing interests

The authors declare that they have no competing interests.

## Authors' contributions

CS and AO analyzed and interpreted the patient data regarding the disease history and were major contributors to the literature review and the critical review of the manuscript. GDA and KDL and TR reviewed the literature and critically revised the manuscript. AO and GDA and PE contributed to the systemic treatment of the disease. JHG contributed to the section on radiotherapy. All authors read and approved the final manuscript.
